# Functional Diversity of Non-Histone Chromosomal Protein HmgB1

**DOI:** 10.3390/ijms21217948

**Published:** 2020-10-26

**Authors:** Elena Chikhirzhina, Tatyana Starkova, Anton Beljajev, Alexander Polyanichko, Alexey Tomilin

**Affiliations:** Laboratory of Molecular Biology of Stem Cells, Institute of Cytology of the Russian Academy of Sciences, 194064 St. Petersburg, Tikhoretsky Av. 4, Russia; t.starkova@incras.ru (T.S.); t800vst1000@mail.ru (A.B.); a.tomilin@incras.ru (A.T.)

**Keywords:** protein HmgB1, DNA-protein, protein–protein interactions, nuclear, extranuclear functions of HmgB1

## Abstract

The functioning of DNA in the cell nucleus is ensured by a multitude of proteins, whose interactions with DNA as well as with other proteins lead to the formation of a complicated, organized, and quite dynamic system known as chromatin. This review is devoted to the description of properties and structure of the progenitors of the most abundant non-histone protein of the HMGB family—the HmgB1 protein. The proteins of the HMGB family are also known as “architectural factors” of chromatin, which play an important role in gene expression, transcription, DNA replication, and repair. However, as soon as HmgB1 goes outside the nucleus, it acquires completely different functions, post-translational modifications, and change of its redox state. Despite a lot of evidence of the functional activity of HmgB1, there are still many issues to be solved related to the mechanisms of the influence of HmgB1 on the development and treatment of different diseases—from oncological and cardiovascular diseases to pathologies during pregnancy and childbirth. Here, we describe molecular structure of the HmgB1 protein and discuss general mechanisms of its interactions with other proteins and DNA in cell.

## 1. Introduction

The functioning of DNA in the cell nucleus is ensured by a multitude of proteins, whose interactions with DNA as well as with other proteins lead to the formation of a complicated, organized, and quite dynamic system known as chromatin. The entire diversity of nuclear proteins can be divided into two distinct groups: histones and non-histone proteins. Five fractions of histones are distinguished by amino acid composition and molecular weight: H1, H2A, H2B, H3, H4. Four of these histones (H2A, H2B, H3, and H4) form a protein complex around which DNA is wound [1,2,3,4,5]. The fifth histone—H1 (in the chromatin of avian erythrocytes exists a variant, called H5)—tightens these DNA–protein complexes together, forming higher levels of chromatin structural organization [3,4,6,7]. 

In the mid-1970s, it was found that a group of proteins with high electrophoretic mobility, called High Mobility Group proteins (hence the name HMG), can be extracted from chromatin together with histones [8]. Further studies revealed that HMG proteins are a structurally and functionally heterogeneous family of architectural and regulatory chromatin proteins [9,10,11,12,13]. It appeared that all HMG proteins bind DNA, regulating structure of chromatin and basic DNA-dependent processes in cell nucleus [14,15]. 

According to their structure and functions, HMG proteins are divided into three families: HMGA (former HMG-I(Y)), HMGB (HMG-1/2), and HMGN (HMG-14/17) [9,14,16]. Proteins of the HMGN family (N stands for nucleosome) bind nucleosomes and contribute to the initiation of transcription [17]. It is important to note that they are not a part of transcriptional complex. It is also known that the competition between HMGN proteins and linker histone H1 for binding AT-rich regions of chromatin affects compaction of DNA [18,19,20]. Proteins of the HMGA family contain a DNA-binding motif, which is known as an AT-hook (hence the name HMGA), these proteins function mainly as dynamic regulators of chromatin structure and transcription of genes [15]. HMGA proteins are involved in various fundamental cellular processes, regulating expression of genes and performing epigenetic regulation. In addition, these proteins are involved in DNA repair, as well as in the processes of cell differentiation and proliferation [21].

This review is devoted to the description of properties and structure of the third, and the most numerous and well-studied family of HMGB domain proteins (from HMG-Box) [3,5,11,12,13,22,23,24,25,26,27,28,29,30,31]. Representatives of this family are actively involved in the structural organization of chromatin and thus play an important role in the regulation of various cellular processes [9,11,12,13,32]. All HMGB proteins have at least one DNA-binding structurally conserved domain, known as HMG-Box, or HMGB-domain [14,22], which is the characteristic property of the proteins within this family. The HMGB domain is a very common motif in eukaryotic proteins [33,34,35,36,37]. It consists of approximately 80 amino acid residues and can be found in a variety of transcription factors, which control differentiation of tissues and determine the sex of an organism during embryogenesis. A distinctive feature of the HMGB motif is the conservative position of hydrophobic aromatic amino acid residues—tyrosine, tryptophan, and phenylalanine—that ensures its strictly defined spatial organization. Different HMGB proteins have different numbers of HMGB domains. Single HMGB domain-containing proteins are characterized by site-specific DNA binding. Single HMGB-domain proteins include such proteins as lymphoid enhancer-binding factor (LEF-1) [33], sex-determining factor SRY and proteins of SOX subfamily [34,35,36,37], chromatin remodeling factors BAF57 and PB1 [10,38,39], and many others. The proteins of this subgroup bind to AT-rich regions in DNA and cause bending of the DNA sequence 5′-(A/T)(A/T)CAAAG-3′ [22]. Examples of multi-domain HMGB proteins, which contain two or more HMGB domains include members of the HmgB1-4 subfamily [10,11,12,13,40,41], mitochondrial factors mtTF1and ABF2 [10], transcriptional factor of RNA-polymerase I UBF [42], drosophila DSP1 protein [43], yeast HMO1/2 proteins [44], and others. Proteins from this subgroup are characterized by non-specific DNA binding. However, they exhibit high affinity to DNA regions with structural anomalies, ranging from bends and kinks of the double helix to supercoiled DNA states and various cross structures, including Holiday junction [45,46], and others.

Among all HMGB proteins, the most common are the progenitors of the family—the HmgB1 and HmgB2 proteins. They are characterized by a high evolutionary conservation of their primary structures: in mice, rats, cows, pigs, and humans these proteins are 95–99% identical [8]. Proteins that are similar to HmgB1 were found in all studied eukaryotes. Similar to the histones H1, the HmgB1 and HmgB2 proteins interact with linker DNA. On average, one HmgB1 molecule can be found per 10–15 nucleosomes [11,47]. The protein appears very stable: its half-life is more than two generations of cells, which is comparable to the half-life of histones [48]. High levels of HmgB1 are generally detected in less differentiated cells [49]. This may explain a high expression of HmgB1 in neoplastic cells which often undergo dedifferentiation [50]. High expression levels of HmgB1 were found in proliferating tissues and actively dividing cells [11]. 

HmgB1 is found in both nucleus and cytoplasm and distribution between these two compartments depends on tissue type, redox state of HmgB1, and the stage of cell differentiation. For example, the thymus, testis, and spleen contain large amounts of this protein, both in nucleus and cytoplasm, whereas brain tissue and liver are mostly characterized by cytoplasmic localization of HmgB1 [51]. It is well known that HmgB1 is actively involved in the regulation of transcription, replication, recombination, and DNA repair [10,11,13,27,52]. However, there is an increasing body of evidence of both cytoplasmic and extracellular roles of the HmgB1, which require systematization and analysis. In this review, we attempted to systematize the variety of extranuclear functions performed by HmgB1 protein.

## 2. Structure of HmgB1 Protein

**Figure 1:**
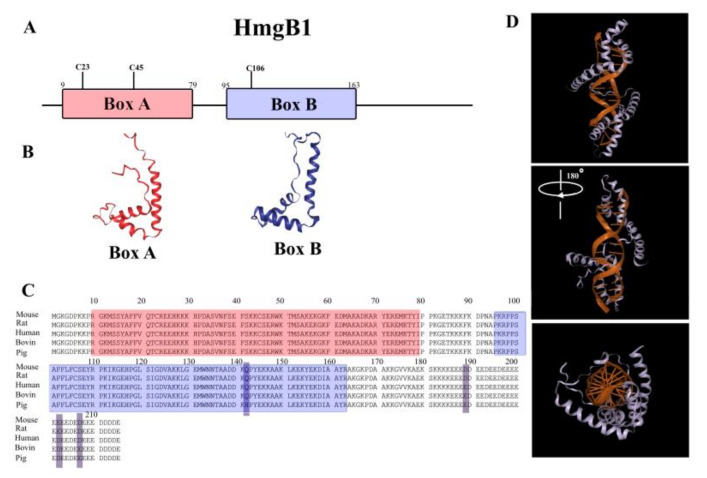
The structure of HmgB1 protein. (**A**) Schematic representation of the structure of chromatin non-histone protein HmgB1. The Box A and Box B are marked by red and blue, respectively; (**B**) the 3D-structure of HMGB domains of HmgB1; (**C**) the amino acid sequence of HmgB1 from mouse, rat, human, bovine, and pig (color scheme as in A); (**D**) The model of interaction of the linked two HMGB domains with DNA. The figure is based on PDB (1AAB, 2GZK) and Uniprot data (P63159; P63158; P09429; P10103; P12682).

In the structure of HmgB1 protein (as well as in the homologous HmgB2), two structurally conserved DNA-binding domains (HMGB-domains A and B), N- and C-terminal regions are clearly distinguished (Figure 1). The C-terminus of HmgB1/2 is represented almost exclusively by sequence of Asp and Glu amino acid residues. Forming a rather flexible structure, this acidic sequence interacts with other parts of molecule [53], having the highest affinity to the B-domain [54]. HmgB1 and HmgB2 differ primarily in the length of this fragment: 30 amino acids in HmgB1 vs. 20 amino acids in HmgB2. The differences in the primary structures of the C-terminal regions are likely to contribute to the differences in the functions of these proteins. It is interesting to note that HmgB1 was identified as an antibacterial secret of human adenoids [55,56] and, according to Gong et al., its antibacterial activity is associated with the sequence of 201–205 aa within the C-terminal region of the protein [57].

According to NMR and X-ray diffraction analysis, approximately 80% of HMGB-domains are in α-helical conformation [14,22,58,59]. There are three α-helices in each HMGB domain (Figure 1), which form a characteristic L-shaped structure with two arms of 31 Å and 36 Å, positioned at the angle of 70–80° to each other [58,59].

This spatial orientation of the α-helical regions is stabilized by hydrophobic interactions between the amino acid residues of helixes I and II in the apex of the corner and the interaction of three proline residues within the N-terminal region with the inner surface of the helix III [60]. This structural pattern is a characteristic property of all HMGB-domain proteins and one can conclude that the proteins of this family are conservative in the structure of their domains, rather than in the amino acid sequence [14,22].

The functionality of HMGB proteins is directly related to their structure. Some HMGB-domain proteins are considered to be “natively unfolded proteins” [11,12,13,15,61]. This group includes proteins which are able to acquire different secondary/tertiary structures upon interaction with different biological molecules. Such variability of the spatial structure of nuclear proteins plays an important role in the formation of their functional complexes in chromatin and in the regulation of chromatin dynamics [62,63].

It was shown that there is a dynamic equilibrium between two different spatial states of the HmgB1—tail-collapsed and extended conformation [53,54,64]. In the collapsed (inactive) state, disordered negatively charged C-terminal region (186–215 aa) is located in the cavity between positively charged DNA-binding A and B domains and interacts with residues of Arg-72 and Arg-162, Lys-81, and Lys-164, as well as with Ile-158 [53], which facilitates stabilization of the domain spatial structure [54,64]. The transition to a functionally active expanded state is accompanied by disruption of these interactions and therefore, by destabilization of the HmgB1 structure. This conformational transition can be induced both by binding to other molecules and post-translational modifications [54,64,65].

## 3. Post-Translational Modifications and Redox State of HmgB1 Protein

**Figure 2:**
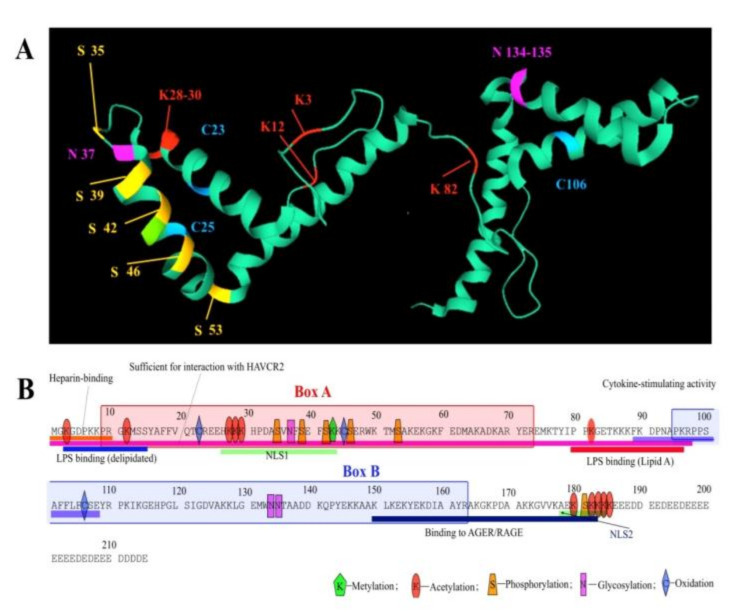
The post-translational modifications of HmgB1. (**A**) The 3D orientation of modified amino acids of HMGB domains of protein; (**B**) Schematic representation of the location of HmgB1 modification sites and functionally significant sections of the amino acid sequence of HmgB1. HAVCR2—Hepatitis A virus cellular receptor 2; LPS—lipopolysaccharide; AGER/RAGE—advanced glycosylation end-product specific receptor; NLS—nuclear localization sequence. The figure is based on PDB (1AAB, 2GZK) and the data are described in the following works [11,65,66,67,68,69,70,71].

Below we have attempted to summarize those sparse data published so far, which describe post-translational modifications (PTMs) of HmgB1 protein (Figure 2). HmgB1 is susceptible to modifications such as acetylation, phosphorylation, methylation, glycosylation, and poly-ADP-ribosylation [11,65,66], which play an important role in the interactions with DNA [67,68,69,70,71] and other proteins [68,72,73], in its nucleus-to-cytoplasm transport [74,75,76,77], and release into extracellular space [78,79,80].

### 3.1. Phosphorylation

Phosphorylation of the HmgB1 primarily regulates its interaction with DNA [11]. In addition, phosphorylation (along with acetylation and methylation) is required for the intracellular localization of the HmgB1. In vertebrates, phosphorylation involves calcium/phospholipid-dependent protein kinase C (cPKC) by the PI3K-PKC signaling pathway [77], but not cAMP-dependent protein kinase [66]. In addition, HmgB1 can be phosphorylated in mouse macrophage cells RAW264.7 and human monocytes after their treatment with phosphatase inhibitors (TNF-α or okadaic acid), leading to HmgB1 translocation to cytoplasm with possible subsequent secretion into the extracellular space [75]. At least six serine phosphorylation sites have been suggested at positions Ser-34, Ser-38, Ser-41, Ser-45, Ser-52, and Ser-180, which are located mainly around the nuclear localization signals (NLS1 and NLS2) of the HmgB1 protein [75]. However, it should be mentioned that neither these sites, nor corresponding kinases involved in the phosphorylation have been accurately identified.

### 3.2. Acetylation

Acetylation is currently the most studied of all HmgB1 modifications. Acetylation affects HmgB1 localization in the cell, its secretion, and binding to DNA. Reversible acetylation was first discovered back in 1979 [81]. Two lysine residues at the N-terminal region of the protein, at positions 2 and 11 are subject to acetylation. It is worth mentioning, that same enzymes are involved in the acetylation of the HmgB1 and histone H4. Lys-2 acetylation affects its ability to recognize structurally damaged DNA regions such as cisplatin adducts, decreasing the anticancer activity of the drug [67]. It was also found that transition of the protein into extended conformation, induced by removal of the C-terminal acidic tail, resulted in acetylation of Lys-81 found on the linker between domain A and domain B [73].

There is no secretory signal peptide in the HmgB1, and the protein does not cross the Golgi apparatus system; therefore, translocation of this nuclear protein into the cytoplasm and further secretion into the extracellular space demands some very specific conditions [74]. Using mass spectrometry, it was shown that in resting macrophages, HmgB1 hyperacetylation triggers the transfer of protein from the nucleus into the cytoplasm. The nuclear localization of HmgB1 is affected by lysine acetylation, particularly within the two major lysine clusters at positions 27–29 within NLS1 and residues 181–183 within NLS2 [74]. It has been demonstrated that mutations of all six lysine residues into glutamines, as a mimic of acetyl-lysine, resulted in partial cytosolic localization [74]. Seventeen lysine residues were acetylated during this transition, which are Lys-27, Lys-28, Lys-29, Lys-179, Lys181, Lys-182, Lys-183, and Lys-184, all of which are located within the NLSs [74].

### 3.3. Methylation

Methylation of Lys-42 in HmgB1 also regulates protein transport from the nucleus to cytoplasm [76]. It was shown that this PTM leads to conformational changes in A domain of HmgB1 weakening its ability to bind DNA. This PTM was only found in neutrophils, although it was suggested that it may also occur in other cells.

### 3.4. Redox State

HmgB1 contains three cysteines (Cys-23, Cys-45, and Cys-106). In the cell nucleus, all three cysteines of HmgB1 are fully reduced. Translocation of HmgB1 protein from nucleus to the cytoplasm is accompanied by oxidation of the Cys23 and Cys45, which form a disulfide bond, whereas Cys106 remains reduced [78]. Upon release of HmgB1 into the intercellular space due to, for example, tissue necrosis the oxidation of the cysteines also occurs. However, in this case the protein is characterized by different set of the PTMs, not typical for secreted HmgB1. The redox status of HmgB1 protein directly affects its extracellular activity associated with immunity and autophagy [78]. At this moment it is well documented that the oxidation of HmgB1 decreases its inflammatory activity both in vitro and in vivo. It is noted that in activated immune cells or damaged cells, translocation to cytoplasm and secretion of HmgB1 into the extracellular space is promoted by reactive oxygen species (ROS) [82]. This means that ROS is one of the main signals that decreases DNA binding activity of HmgB1 in the nucleus and promotes its cytoplasmic localization and further secretion by the cell. Depending on the total modification status, partially oxidized HmgB1 can interact with a number of receptors, taking part in various cellular processes. The redox state of the protein can also affect its ability to bend DNA [83] and interact with non-canonical DNA structures [84]. The role of HmgB1 outside the nucleus will be further discussed in the corresponding section of the review.

### 3.5. Glycosylation

HmgB1 protein can be N-glycosylated at asparagine residue at position 37 (Asn-37) and, alternatively, at Asn-134 or Asn-135 residues. The two N-glycosylation sites, Asn-37 and Asn-134 (unlike Asn-135), are located in the consensus for N-glycosylation motifs of Asn-XXX-Ser/Thr (where XXX is any amino acid except proline) [85]. It is shown that PTMs of acetylation, phosphorylation, and oxidation are not sufficient for HmgB1 secretion in intercellular space. Nuclear HMGB1 is translocated to the cytoplasm after binding to the nuclear exportin CRM1 [74]. It was shown [85], that N-glycosylation of HmgB1 is important for binding with CRM1. N-glycosylation of HmgB1 is mediated by phorbol 12-myristate 13-acetate (PMA), trichostatin A (TSA), and lipopolysaccharide (LPS) and can lead to the secretion of the protein into the extracellular space as a result of decreasing HmgB1-DNA binding affinity and increasing association with nuclear export protein CRM1 [85]. Thus, N-glycosylation of HmgB1 is a pre-requisite for its nucleus to cytoplasm translocation and extracellular secretion.

### 3.6. ADP-Ribosylation

Similar to the HmgB1 modifications described above, ADP-ribosylation affects localization of the protein within the cell. Mono and poly-ADP-ribosylation are necessary for nuclear export and release of HmgB1 during cell death, especially during necrosis [66,86,87]. Furthermore, these HmgB1 modifications lead to overall suppression of gene transcription [66]. ADP-ribosylation affects the binding of HmgB1 to RAGE (receptor for advanced glycation end products) [88], increasing the inhibition of dead neutrophil uptake by macrophages—the process of efferocytosis. Moreover, a decrease in the amount of intracellular HmgB1 leads to excessive activation of the poly-ADP-ribose polymerase 1 (PARP1) [89]. PARP1 promotes repair of damaged bases and single-stranded DNA breaks by modulating the structure of chromatin and binding DNA repair factors. Thus, there is a cross-link between ADP-ribosylation of HmgB1 and PARP1 in regulating cell death.

It is worth mentioning that the biological functions of the majority of PTMs identified up to date remain unknown. However, even from the available data, it is clear that the modification status of the protein, together with its redox state, is very important determinants of the cellular localization of the protein. These PTMs are parts of a mechanism that regulates HmgB1 participation in certain cellular and extracellular processes.

## 4. Functions of HmgB1 Protein in Cell Nucleus

### 4.1. Interaction with DNA

HmgB1 is known primarily as a nuclear protein that, like histone H1, interacts with the internucleosomal (linker) DNA regions [5,11,27,90]. Binding of HmgB1 to linker DNA leads to a significant bending of the double helix at the binding site. Investigation of the plasmid DNA-HmgB1 interactions by optical and hydrodynamic approaches revealed that binding of the protein to DNA resulted in local changes of the double helix geometry in the vicinity of the binding site [9,11,31,91,92]. Local changes in DNA geometry facilitates functioning of other proteins and their complexes on DNA, including different transcription factors [11,13,93]. All proteins from the HMGB family are characterized by very high affinity and selectivity to DNA regions with various structural abnormalities, such as 4H DNA, bends and crossovers, as well as single- and double-stranded breaks [11,46,71,94]. HMGB partly intercalates into the minor groove of DNA by its aromatic amino acid residues inducing bending of the helix toward the major groove. The bend in angle may reach 140° [9,11].

HmgB1 and other sequence nonspecific HMGB domain proteins (such as ribosomal transcription factor UBF) [42] in presence of topoisomerase I, are able to change the degree of helicity in topologically closed regions of DNA. These proteins can form of loops in linear DNA molecule and in circular plasmids even in the absence of other factors. Thermodynamic data [95] show that in most cases only B-domain of the protein is involved in the interaction with DNA (single domain binding). However, some authors suggest, that both DNA-binding domains participate in the interactions with AT-rich regions (two-domain binding) [95]. In eukaryotes, AT-rich regions are mainly located in the promoter regions of the gene at a distance of 30 nucleotides above the transcription initiation point [96].

Physico-chemical studies of the DNA–HmgB1 complexes demonstrated that local concentration of the protein can significantly affect the structure of resulted complex, and excess of the HmgB1 induces numerous protein–protein interactions facilitating formation of structurally ordered supramolecular complexes [23,24,28,31,92,97]. It was also shown that C-terminal tail of the protein regulates DNA binding activity of the protein [28,54,60,97,98]. Deletion or inactivation of the acidic tail resulted in simultaneous binding of both HMGB-domains to DNA, even if normally only one of the HMGB-domains is involved in DNA–protein complex formation [60,97,98].

### 4.2. Interaction of HmgB1 with Other Proteins

Over the past decades, a number of studies were focused on the interaction of HmgB1 with other proteins [12,83]. It is commonly accepted that the main function of HmgB1 is to modulate chromatin structure, and hence the histone proteins are the major partners of HMGB proteins in chromatin. It has been shown that HmgB1 binds both core histones (for example, histone H3 [64]) and linker histone H1. The binding occurs via flexible N-terminal region of histone and disordered C-terminal tail of HmgB1. Direct interaction between H1 and HmgB1 has been reported by different methods, including circular dichroism UV and IR regions [72,99,100], atomic force microscopy [29], and fluorescent approaches [101]. It was revealed that the structure of at least one of the proteins changed upon the interaction [101]. The possibility of interaction between the C-terminal regions of histone H1 and HmgB1 protein has also been shown using small-angle X-ray scattering and NMR [72]. However, it is still unclear how the interaction between histone H1 and HMGB-domain proteins affects the structure and biological functions of genome. According to some authors, HmgB1 is able to displace H1 from its binding site to facilitate the sliding of nucleosome along the DNA, which is essential for the binding of transcription factors [12,72,83]. According to this model, negatively charged C-terminal sequence of HmgB1 binds a positively charged N-terminal region of histone H1, disrupting its interactions with DNA and leading to the displacement of H1. DNA bends formed by HmgB1 facilitate formation of loops required for the initiation of binding of the remodeling complex, which in turn is responsible for the sliding of the nucleosome, until the transcribed region of DNA becomes available for transcription factors. Next, the process of HmgB1-dependent binding of transcription factors to DNA takes place.

**Figure 3:**
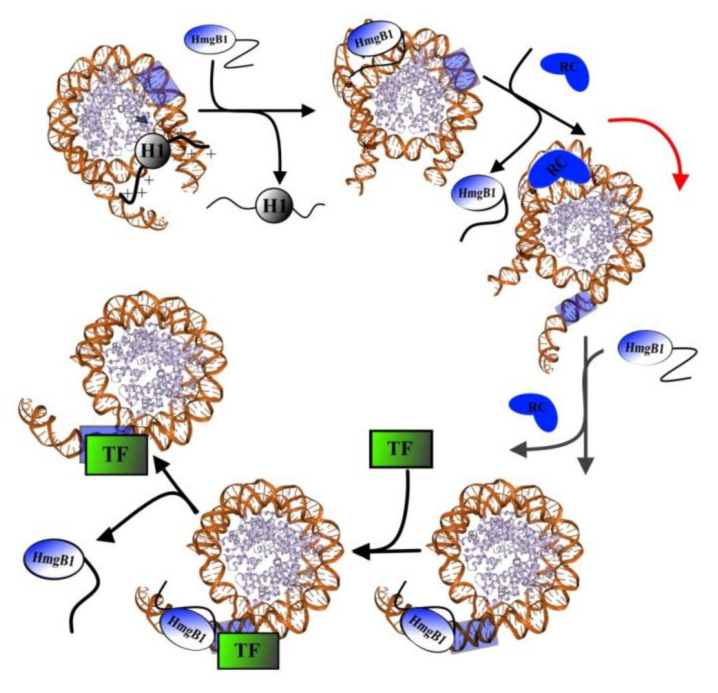
The model of the sliding of nucleosome along the DNA. According to this model, the binding of HmgB1 with H1 histone results in disruption of H1 interactions with DNA and its displacement. The loops of DNA formed by HmgB1-DNA binding are necessary for interaction of the remodeling complex (RC) with DNA. In turn, PC–DNA complex is responsible for the sliding of the nucleosome, until the transcribed region of DNA becomes available for transcription factors (TF). Next, the process of HmgB1-dependent binding of transcription factors to DNA takes place.

It was suggested that the interaction of transcription factors with DNA might occur via the formation of an intermediate complex TF1–DNA–HmgB1, whose formation induces attachment of other regulatory chromatin proteins [11]. It should be noted that according to this model, the bending of the DNA double helix itself during the DNA–HmgB1 interaction is not a signal for TF attachment. Only direct interaction of TF with HmgB1 results in TF binding to DNA (Figure 3).

Molecular mechanisms of transcription initiation or activation in vivo are not clear yet. Investigations show, that histone H1 acts as repressor of transcription, while HmgB1 protein can act as the main factor that stimulates or, depending on the conditions, reversibly inhibits the hormonal activation of RNA transcription by polymerase II [101,102,103]. It is likely that H1 and HmgB1/2 proteins, when bound to DNA, perform a common function. Meanwhile, the experimental data currently available are somewhat contradictory. Some authors suggest that HmgB1 can compete with histone H1 for binding to local chromatin regions, affecting their functional activity [9,11,104]. At the same time, other researchers report that these proteins can act as partners [103]. Despite the fact that H1 and HmgB1 interact within different grooves of DNA (major and minor, respectively), both proteins change the structure of the double helix in a similar way: positively charged H1 screens the charges of phosphate groups decreasing the rigidity of the double helix and bending it toward the major groove. In turn, HmgB1 partly intercalates with its aromatic amino acid residues into the minor groove of DNA, inducing bending of the helix, also toward the major groove [9]. Some authors suggest that binding one of these two linker proteins to DNA stimulates binding of the other [26,27,29,103,105,106,107,108].

Positively charged histone H1 interacts not only with the phosphate groups of DNA but also with the negatively charged C-terminal domain of HmgB1 [29,30]. Analysis of such complexes using atomic force microscopy revealed the formation of fibril-like structures formed by several DNA molecules and stabilized not only by DNA-protein, but also by protein–protein interactions [72,108,109]. Thus, HmgB1 functions primarily as an architectural protein in chromatin [11,110].

Along with histone H1, HmgB1 protein may modulate the integrity and the compactness of chromatin fibers. Earlier it was reported that HMGB-domain proteins might participate in nucleosome assembling in vitro [9,10,11,111]. Some mononucleosomes from the total chromatin can contain sufficient amounts of HmgB1 (and HmgB2), but not of histone H1. In vitro, HmgB1, like histone H1, is able to induce a chromatosomal pause during digestion of chromatin by micrococcal nuclease [112]. These data suggest that the main role of the HmgB1 is to replace histone H1 in the linker region to facilitate thereby accessibility of local chromatin domains [9].

In addition to the structural functions, HmgB1 protein is also involved in all repair processes. It participates in formation of a mismatch repair (MMR) multi-protein complex during DNA replication [13]. HmgB1 works during the initial stages of recognition/damage of the DNA and interacts with MMR proteins, such as Hsp70, MSH2, and MLH1 [113,114,115]. Base excision repair (BER) and nucleotide excision repair (NER) are also associated with the interaction between HmgB1 and various enzymes [67,68,69,70,116,117,118]. Repair of double-strand breaks (DSBR) involves DNA-dependent protein kinase (DNA-PKcs) [119]. It was demonstrated that in vitro HmgB1 protein stimulates activity of DNA-Pkcs [119], RAG1 and RAG2 proteins [120] and increases the activity of T4 DNA ligase [121]. Upon recombination the complex between RAG1, RAG2, and HmgB1 proteins induces the formation of hairpin on DNA [122]. Moreover, this protein complex is stable during the entire process of recombination. Also, the presence of HmgB1 activates the 5′-dRP lyase which is involved in the repair of single-strand breaks.

At the same time, HmgB1 can inhibit DNA repair, for example, in the case of the interaction with platinum adducts on DNA [41,94,123], which are formed due to change of the local structure of the double helix in DNA [45,124,125,126] during its binding with some antitumor drugs such as cisplatin and other platinum coordination compounds.

It was shown that in this case, HmgB1 inhibits DNA repair after damage resulted from formation of stable DNA–cisplatin–HmgB1 complex, which might affect tumor susceptibility to chemotherapy [123]. Analysis of the nucleic acid repair performed in vitro revealed that this process was slowed down in the presence of HMGB-domain proteins [127]. Probably, HMGB-domain proteins protect DNA regions damaged by cisplatin, preventing access of the repair complexes. It was shown, both in vivo and in vitro [128,129], that the presence of these proteins increases the efficiency of cisplatin treatment. Therefore, HMGB-domain proteins can be considered as potential therapeutic drug targets for the treatment of tumors and other diseases [127].

Another partner of HmgB1 is a tumor suppressor p53 protein often called the “genome keeper”, which has regulatory roles in different processes, such as DNA repair, metabolism, cell cycle arrest, cellular senescence, autophagy, and apoptosis [130]. Interaction of HmgB1 with p53 was demonstrated both in vitro, and in vivo [131]. HMGB-induced DNA bent facilitates binding of p53 to DNA. In the case of DNA damage, HmgB1 can act as a chaperone stimulating interaction of p53 with specific DNA sites and subsequently leaving the complex [132,133] and regions damaged by cisplatin [134]. It was shown that p53 interacts with the A-domain of HmgB1 and that this interaction is regulated by C-terminal tail of HmgB1 [131]. In addition, it was shown that HmgB1/p53 complex regulates apoptosis and autophagy [135,136].

### 4.3. Effect of Expression Levels of HmgB1 and HmgB2 on Mouse and Human Stem Cell Chromatin, Mouse Embryogenesis, and Postnatal Development

According to published data, self-renewal and differentiation of embryonic stem cells (ESCs), as well as growth rate of malignant tumors in animals, directly depends on the expression levels of *HmgB1* and *HmgB2* genes. It was shown that a decrease in the expression of *HmgB1* and *HmgB2* (both individually and simultaneously) in human ESC chromatin does not significantly affect to the morphology of the cells and their pluripotent status [137]. However, it was shown that knockdown of the *HmgB1* and *HmgB2* genes separately leads to a change in the rate of proliferation of ESCs and cell cycle disorders. Knockdown of the *HmgB2* gene promotes the transition of cells to the S and G2/M phases of the cell cycle, while knockdown of the *HmgB1* gene leads to a delay in the G1 phase [137].

A decrease of proliferation rate in case of knockdown of the *HmgB1* gene was also observed in a number of cancer lines having HmgB1 knocked down. For example, it was shown that HmgB1 knockdown can specifically and efficiently inhibit cell proliferation, migration, and invasion, induce apoptosis, and cell arrest of bladder and glioma cancer cells at the G0/G1 phase [138,139].

It was further shown that HmgB1 knockdown in mouse nerve stem cells can inhibit the phosphorylation of MAPK family proteins, such as ERK, JNK, and p38, which are involved in cell cycle regulation and thus can influence cell proliferation [138,140].

Along with the absence of significant changes in the phenotype of human ESCs in the case of simultaneous knockdown of the *HmgB1* and *HmgB2* genes, no significant deviations were found in the study of telomerase activity in these cells [137]. However, decreased level of *HmgB1* gene expression in differentiated cells leads to decreasing telomerase activity and telomere dysfunction. However, using quantitative PCR analysis, other authors demonstrated that knockdown of the *HmgB2* gene in mouse ESC chromatin (line J1) reduced the expression level of majority of the genes regulated by Oct4, including pluripotency associated genes *Oct4, Sox2, Nanog, PcG Phc1*, and DNA damage response genes *Parp1*, *Brca1*, and *Trp53* [141]. It is important to note that the expression level of protein kinases B, which regulate cell proliferation (total Akt, activated Akt, and totally phosphorylated Akt substrates), also decreases in the case of knockdown of the *HmgB2* gene in embryonic stem cells, which confirms the relationship between Oct4, Akt, and HmgB2 proteins [141].

Differences in phenotypes of human and mouse ESCs during knockdown/knockout of *HmgB1* and *HmgB2* might be due to the fact that cultured human and mouse stem cells are at different stages of the pluripotent state [142]. Mouse ESCs belong to the so-called naive pluripotent cells and correspond to the pre-implantation period of the blastocyst. At the same time, human ESCs are in the primed state, which corresponds to cells of epiblast shortly after implantation. Naive and primed pluripotent stem cells differ in expression patterns of a number of genes [142]. The presence of the classic pluripotency markers Oct4 and Sox2 plays similar role in human and mouse ESCs, but the regulators working further down the signaling pathway seem to be not so conservative. At the same time, the difference between cell phenotypes in the cases of knockdown and knockout of *HmgB1* and *HmgB2* genes can be explained by the ability of the cells to compensate nonsense mutations by increasing expression of homologous proteins from closely related genes, which are able to fully or partially compensate for the function loss during the knockout (but not the knockdown) [143,144].

It was shown that knockdown of the *HmgB1* gene leads to an increased probability of the apoptosis development in both ESCs and human neuroectodermal cells [145,146]. Moreover, this effect is most prominent in differentiated cells. The cause of apoptosis in the embryonic brain of HmgB1 KO mice might be due to the impaired cytosolic signaling of HmgB1/p53 in neuronal progenitors [135]. It is known that knockout of the HmgB1 leads to the cytosolic localization of p53, resulting in inhibition of autophagy [135]. Moreover, decrease in level of HmgB1 expression leads to disruption of evolutionarily conserved Wnt/β-catenin signaling pathway, which regulates the cell migration, cell polarity, organogenesis during embryonic development [146]. At the same time, it was found that the overexpression of Wnt/β-catenin may lead to the accumulation of p53 protein in the cancer cell [147]. Thus, the excessive expression level of Wnt/β-catenin in the mice lacking HmgB1 might be one of the major reasons of the apoptosis and reduced proliferation of neuronal cells [148].

In a culture of immortalized murine embryonic fibroblasts (MEF), it has been shown that knockout of the *HmgB1* gene leads to chromosome instability and an increased number of chromosome aberrations [145]. These chromosomal disruptions likely result in apoptosis of the culture of differentiated cells with decreased HmgB1 expression. Importantly, in case of a HmgB1 knockdown, apoptosis occurs only in cells expressing HmgB2 at a normal level. In case of deficiency of both HmgB1 and HmgB2 proteins, ESCs and human neuroectodermal cells do not differ from control cells [137].

Teratoma assay (teratomas were obtained by injecting human ESC with HmgB1 or HmgB2 knockdown into OD/SCID/IL2Rg-null mice) did not reveal significant problems of the differentiation process itself: teratomas contained tissues of all three germ layers (ectoderm, endoderm, and mesoderm). At the same time, in the both cases, a significant change in the rate of teratomogenesis was observed, which corresponds to the proliferation rate of each of the cell lines [137].

Newborn mice with *HmgB1* knockout showed a defect in the eyelids, low motor activity, and a high lethality rate on the first day after birth [149]. The cause of lethality of these mice is hypoglycemia, which was compensated during the prenatal period of development by glucose intake through the placenta. Mice which received an intraperitoneal injection of glucose every 6 h after birth remained viable. However, the postnatal development of *HmgB1^−/−^* mice indicates serious problems during embryogenesis. In addition to blindness, *HmgB1* knockout mice were characterized by a decrease in growth rate, the presence of ruffled hair, an anomaly in the structure of the hind limbs, and impaired posture. *HmgB1^−/−^* mice did not survive to reproductive age even with persistence of glycotherapy. At the same time, *HmgB2* knockout mice appear to be somatically healthy and maintain an average lifespan [149]. It was shown that the *HmgB1* knockout mice are characterized by a decreased expression of BMP and TGF-β cytokines, which may lead to a reduced size of embryos and delayed post-natal development. In addition, *HmgB1* knockout mice have shown defects of brain development due to decreased expression levels of the growth factors BDNF, FGF2, GDNF, AMIGO1, and ACHE, which are important for the formation of neuronal connections [146]. However, it was later shown that knockout of *HmgB2* gene can also lead to impaired like impaired spermatogenesis and fertility in male mice [150]. *HmgB2^−/−^* spermatozoa are motionless, have a shortened flagellum and a deformed head shape. Degeneration of germ cells in the seminiferous tubules was also detected. The in vitro efficiency of the formation of a zygote with the participation of these defect spermatozoa did not differ from the efficiency of fertilization in the control experiments [150]. However, under natural conditions, the revealed defects of spermatogenesis are likely to lead to impaired of reproductive function in HmgB2 deficient mice.

In addition to the abnormalities in reproductive system, it was found that the heart of one-year-old *HmgB2^−/−^* mice was characterized by systolic dysfunction of the ventricles. Histological analysis of the heart tissue of these mice revealed an increased number of cardiomyocytes and the presence of fibrous regions. Using quantitative PCR analysis of heart tissue, an increased, as compared to the wild-type control, levels of HF, Bnp, and Myh7 markers, as well as of the fibrosis markers Collagen1 and Ctgf were revealed, which indicates premature aging of heart cells with HmgB2 deficiency [151].

## 5. Extranuclear Functions of HmgB1

**Figure 4:**
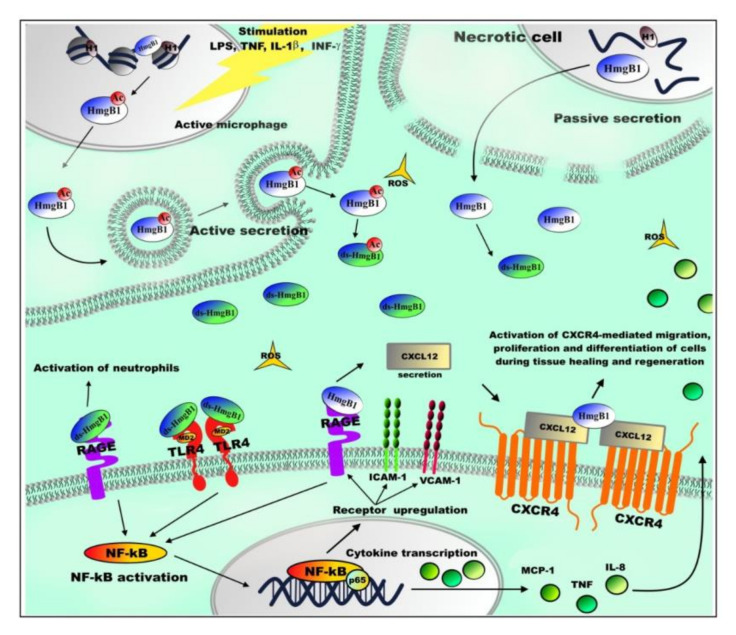
Extranuclear functions of HmgB1.The tumor necrosis factor (TNF), lipopolysaccharide (LPS), or interferon gamma (INF-γ) stimulation leads to acetylation of HmgB1 in NLS regions and results in the transport of protein from nucleus to cytoplasm and extracellular space. In the extracellular space the HmgB1 is oxidized to ds-HmgB1 by reactive oxygen species (ROS). The functions of ac-ds-HmgB1 are still unclear. In case of disturbance of cell integrity, non-acetylated HmgB1 is released to the extracellular space. Depending on the REDOX state, HmgB1 can act as signal molecule (by activation the signaling pathways of MAPKs, NF-kB, and phosphoinositide 3-kinase/AKT), take part in regulation of cell migration, or participate in immune response and in the synthesis of anti-inflammatory cytokines.

The variety of functions performed by HMGB1 protein can be further illustrated by the fact that the protein was found in nucleus, cytoplasm, and extracellular space [11,13,52,152,153]. Protein localization depends on the pattern of its post-translational modifications (PTMs) [74,76,77,154], as well as the redox state of cysteine residues [52,78,80]. HmgB1 has two regions of nuclear localization signals (NLS1 and NLS2) and two nuclear export signals (NES), whose presence implies its ability to migrate between the nucleus and cytoplasm [74]. Nuclear localization of HmgB1 requires deacetylation of lysines in NLS1 and NLS2 regions by sirtuin-1 (Silent Information Regulator Two 1, SIRT1) [155]. Acetylation in NLS regions leads to HmgB1 binding to receptors responsible for its transport from nucleus to cytoplasm [74,80] (Figure 4). However, it is still unclear how acetylation of HmgB1 in NLSs affects its extra nuclear functioning. The translocation of HmgB1 from/into nucleus is controlled by serine/threonine phosphorylation [65,77], and in neutrophils—by methylation of K42 [76]. Additionally, it is worth mentioning that the release of the protein from nucleus into the cytoplasm can occur as a result of cell damage due to pyroptosis [156], apoptosis [157], or necrosis [158]. The patterns of HmgB1 PTMs are very different in the cases of protein release from nucleus during necrosis and “signal” translocation of the protein into the cytoplasm [65,74,76,80].

The extracellular activity of HmgB1 is influenced by the REDOX state of the three conserved cysteines: C23, C45, and C106 [78,79,159]. In cell nucleus, all three of the cysteine residues are fully reduced (fr-HmgB1). fr-HmgB1 take part in the transcription, repair, recombination, and replication of DNA, acting as DNA-chaperone (Figure 2) and recruiting the chromatin remodeling complex to nucleosome and facilitating the binding of transcription factors to DNA [133,160].

In case of necrosis or other disturbance of cell integrity, non-acetylated HmgB1 is released into the extracellular space (Figure 3), where it is oxidized by reactive oxygen species [161]. HmgB1 can be oxidized to the disulfide form (ds-HmgB1), which is characterized by the formation of a disulfide bond between C23 and C45 and the presence of C106 in the reduced state, and further to sulfonic form (ox-HmgB1) when oxidation of all three of the cysteine residues occurs [78]. The half-life of fr-HmgB1 before it becomes converted into ds-HmgB1 varies from 17 min in blood serum in vitro to 3 h in cell culture medium [162]. Depending on the oxidation level, HmgB1 can (a) act as signal molecule (by activation the signaling pathways of MAPKs, NF-kB and phosphoinositide 3-kinase/AKT), (b) take part in regulation of cell migration, or (c) participate in immune response and in the synthesis of anti-inflammatory cytokines [52,78,79,159,163,164,165,166].

It is found that the redox state of HmgB1 is tissue-specific. The normal spleen and liver are characterized by a high level of ds-HmgB1, while in muscle the level of ds-HmgB1 is very low and only appears after acute damage. Similarly, levels of ds-HmgB1 are high in the tumor microenvironment, at the same time it is absent in cachectic muscles from the same tumor bearing mice [167]. It has been suggested that the tissue itself determines the redox state of the extracellular space and, thereby, the oxidative state of the released HmgB1 [162]. The presence of ds-HmgB1 has been shown to be closely related to tissue inflammation, and leukocytes are thought to be the main source of ds-HmgB1 [167].

It has been demonstrated that the receptor for advanced glycation end products (RAGE) is the main partner of extracellular HmgB1 at transmitting immune response signals [168,169,170]. fr-HmgB1 can also bind to RAGE and promote autophagy by inhibiting mammalian target of rapamycin (mTOR) kinase and promoting Beclin1–Ptdlns3KC3 complex formation [171]. It was shown that ds-HmgB1 binds RAGE with a higher affinity [164,165]. RAGE binding to fr-HmgB1 stimulates production of CXCL12 (C-X-C)-motif chemokine 12). The interaction between fr-HmgB1 and CXCL12 protein results in the HmgB1 binding to the receptor CXCR4 (C-X-C chemokine receptor type 4) and activation of CXCR4-mediated migration, proliferation, and differentiation of cells during tissue healing and regeneration [52,172]. At the same time, the interaction of RAGE with ds-HmgB1 (ds-HmgB1 binds RAGE with a higher affinity compared to fr-HmgB1 [164,165]) leads to activation of neutrophils and the formation of their extracellular traps, which is important in thrombotic and inflammatory processes [164]. Experiments with animals demonstrated that the inhibition of HmgB1 interaction with this receptor suppresses tumor growth and spread metastasis [173,174,175]. It was shown that the interaction of HmgB1 with RAGE receptors on the cell surface can lead to MAPK activation and thus promote the TLR4 translocation on the cell surface [176].

ds-HmgB1 interacts with TLR4–MD2 complex (Toll-like receptor 4 (TLR4)/myeloid differentiation factor 2 (MD-2)). This interaction stimulates release of inflammatory and angiogenic factors by activation of transcription nuclear factor NF-κB. The interaction of HmgB1 with TLR2 or TLR4 regulates inflammation process when lungs or liver are damaged due to epilepsy, heart disease, or cancer [52]. For example, the formation of the HmgB1–TLR4 complex stimulates synthesis of cytokines and chemokines in inflammatory cells [52,78,79,159,163]. The HmgB1 binding to TLR2 leads to activation of the so-called “natural killers” [177,178]. In addition, it is known that extracellular HmgB1 can bind to single-stranded oligonucleotides by forming an HmgB1–5′-C-phosphate-G (CpG)–DNA complex and, as part of this complex, the protein interacts with TLR9, increasing cytokine production [168]. HmgB1 can also interact with B-cell differentiation marker CD24 and reduce the inflammation during tissue damage by CD24–Siglec-10 complex formation, which negatively regulates NF-κB [179].

From a functional point of view, further oxidation of HmgB1, in which all three cysteines are oxidized [78], is less studied. According to some authors [52] it is possible that the protein in this state is functionally inert. Some studies revealed [180] that ox-HmgB1 is found in the late stages of inflammatory processes and it is associated with tissue regeneration and activation of neutrophils [79]. On the contrary, later it was demonstrated that the fr-HMGB1 is involved in tissue regeneration [172].

The extracellular functions of HmgB1 allow one to classify it as an alarmin [52,152,153,166,181,182,183,184]. Alarmins are synthesized during inflammatory processes and activate cells of the immune system, which ultimately contributes to the restoration of damaged tissue [79,152,153].

Overall, extracellular HmgB1 promotes wound healing and tissue regeneration [165]. HmgB1 affects migration of mesenchymal stem cells, and its use in cell therapy has been shown to promote the synthesis of small cytokines—chemokines which increase the rate of stem cell migration [52,78,79,159,163,185]. Similar experiments with animals show that inhibition of extracellular HmgB1 leads to a decrease of inflammatory processes, ameliorating the course of diseases [52]. 

Major proteins involved in interactions with HmgB1 described above are briefly summarized in Table 1.

### 5.1. HmgB1 and Bioenergetics of Eukaryotic Cells

Mitochondria play a major role in the bioenergetics in majority of eukaryotic cells [186]. *HmgB1* knockout mice are known to die shortly after birth from severe hypoglycemia [150]. This circumstance may be an evidence of the disruption of main cellular processes. Indeed, it has been shown that the basal level of both oxidative phosphorylation (OXPHOS) and glycolysis of mouse embryonic fibroblasts (MEFs) knocked out by the *HmgB1* gene was significantly reduced [187]. The respiratory dysfunctions of mitochondria are attributed to their morphological features. In differentiated eukaryotic cells, mitochondria are elongated oval organelles. Analysis of the structure of mitochondria in cells with a knockout of *Hmgb1* gene showed that deficiency in HmgB1 protein resulted in formation of shortened and rounded mitochondria, which lost their functionality [188] Increased level of HmgB1 in MEFs with *Hmgb1* knockout due to the expression of its exogenous form leads to the restoration of glycolysis, mitochondrial morphology, and, as a consequence, their respiratory functions [187]. Similar effects were observed in case of knockdown of the *HmgB1* gene.

The decrease of HmgB1 expression by RNA interference in MEFs, NIH-3T3 mouse fibroblasts, Panc2.03 human pancreatic cancer cells, and HCT116 human colon cancer cells resulted in a decrease in OXPHOS level. Cells with HmgB1 knockdown showed 20–40% decrease in basal ATP levels, which is accompanied by decreased cell growth and proliferation. It was also shown that a decrease of HmgB1 expression did not affect the total mitochondrial mass of the cell [187].

One of the partners of HmgB1 protein is the heat shock protein beta-1 (HSPB1, also known as HSP25 in mice and HSP27 in humans). HSPB1 is expressed in various types of cells and tissues. Knockout of the *HSPB1* gene leads to lethality of mice. This circumstance indicates that HSPB1 is required for growth and embryonic development [188,189]. It was shown that a decrease in the expression of HmgB1 in MEFs leads to a significant decrease in the level of expression of HSPB1 [187]. At the same time, there is a decrease of the rate of oxygen consumption, extracellular acidification of the medium, ATP production, and increased fragmentation of mitochondria. The observed effects are surprisingly similar to those observed in cells with deficiency in HmgB1 protein [187]. At the same time, increasing level of HSPB1 in *HmgB1^−^*^/*−*^ cells to physiological level by expression the exogenous form of HSPB1 leads to restoration of the mitochondrial structure, mitochondrial respiration and ATP production. In this regard, it can be assumed that the observed increase in number of dysfunctional mitochondria in cells with HmgB1 deficiency is associated with the loss of one of its partners, namely HSPB1.

Autophagy is an important biological mechanism for eliminating dysfunctional or senescent macromolecules and organelles [170,190]. Mitophagy is responsible for the destruction of dysfunctional or structurally disordered mitochondria [191,192]. Mitophagy involves the formation of autophagosomes to absorb dysfunctional mitochondria, the fusion of lysosomes with autophagosomes, and the degradation of dysfunctional mitochondria by autolysosomes.

Cellular stress is one of the causes of mitochondrial damage. During this process, stress fibers are formed, which are bundles of actin filaments. The level of co-localization of mitochondria and actin increases, which seems to be an important regulatory mechanism for the restoration of mitochondrial damage. A well-known function of HSPB1 is associated with its interaction with actin cytoskeleton [193,194]. The actin cytoskeleton is a dynamic structure that maintains the shape of cells and plays an important role in the transport and morphology of intracellular vesicles and organelles, including mitochondria [195]. It has been shown that the actin polymerization and reorganization are modulated by HSPB1 expression level [194,196].

A decrease in the level of HSPB1 expression, in turn, reduces the efficiency of stress fiber formation and inhibits the co-localization of mitochondria with actin, autophagosomes (LC3, microtubule-associated protein 1 light chain 3), and autolysosomes (LAMP2, lysosomal-associated membrane protein 2) [187]. Since decreasing level of HmgB1 expression leads to a deficiency in the HSPB1 protein, a deficiency in autophagy and, therefore, an increase in the number of dysfunctional mitochondria and mitochondria with structural disorders become evident [187].

### 5.2. HMGB1 and Cellular Senescence

As it was mentioned above, HmgB1 is involved in many cellular processes, and in cellular senescence in particular. Cellular senescence is usually associated with the replicative aging, when a cell loses its ability to divide. Cellular senescence is characterized by a decreased intensity of energy exchange and rate of RNA and protein synthesis, decreased efficiency of DNA repair, and increased level of accumulation of mutations. A disbalance in cellular population is often observed, which occurs due to the fact that individual cells enter into the senescence not simultaneously.

Decreased functional activity of cells due to time course also refers to the cellular senescence [197,198]. HmgB1 depletion in the nucleus was observed in 50% of senescent cells, as a result of the translocation of the protein into the cytoplasm and further secretion into the extracellular space [181,199] The most prominent HmgB1 depletion is observed in senescent cells with large nuclei (regardless of cell line). Noteworthy, cells lacking nuclear HmgB1 are characterized by a decreased number of nucleosomes, making chromatin more susceptible to DNA damage and false transcription [200,201,202,203].

Cells with low levels of HmgB1 expression are characterized by an increase of transcription level of genes associated with senescence-associated secretory phenotype (SASP). These genes include genes of growth factors, proteases, and inflammatory cytokines that act paracrine on neighboring cells [181]. To identify possible HmgB1-associated protein complexes or complexes that can replace HmgB1 in the chromatin of senescent cells, Sofiadis with colleges mapped HmgB1 binding genome wide [199].

It was found that the binding sites of the transcription factors of the E2F family, which take part in the control of the cell cycle and the functioning of tumor suppressor proteins, are significantly enriched with HmgB1 protein. In addition, the binding sites for senescence activating co-repressors (for example, REST and HEY2) and the architectural protein Zinc finger and BTB domain-containing protein 7B (ZBTB7B), which plays an important role in the activation of inflammatory genes, are also enriched with HmgB1 protein [204]. Nuclear depletion of HmgB1 leads to a significant HMGB-deficiency at the corresponding chromatin sites, contributing to transcription regulation. Thus, the basal level of HmgB1 expression prevents premature senescence of the cells.

## 6. HMGB1 and Structural Organization of Genome

Topologically associated domains (TADs) are genomic regions with strong spatial localization of intermolecular interactions. These regions are often marked by the presence of CTCF (CCCT-binding factor) and/or active gene promoters [205]. It has been shown that a significant number of TAD boundaries in proliferating human cells are marked by HmgB1. TADs of senescent nuclei are characterized by a loss of delimitation, and the boundaries marked with HmgB1 demonstrate loss of isolation and characterized by different pattern of spatial interactions. Thus, it becomes clear that the loss of HmgB1 during aging contributes to TAD remodeling [199].

Human chromosomes show significant changes of structure during replicative arrest [206]. These changes in chromosomes are more prominent in deep cellular senescence [207]. Considering TADs as the functional blocks of chromosomes [208], one might wonder how topologically associated regions can spatially associate to produce mega-TADs [209] and other functional topologies. During cellular senescence, increasing number of clusters, including several consecutive TADs, were observed, indicating the fusion of TADs. The fusion of TADs is consistent with the theory of spatial densification of chromatin during senescence [206,207]. It should be noted that “single” TADs were also identified, usually located between clusters of several TADs [199]. Surprisingly, the boundaries of these single TADs were marked by HmgB1 to a greater extent, compared to the boundaries of clusters consisting of three or more consecutive TADs. It was found that the clusters with multiple TADs contain genes involved in regulating the structural organization and conformation of chromatin. Genes, that are unique to single TADs, are associated with SASP production and its subsequent effects.

It has been suggested that the loss of HmgB1 in the nuclei of senescent cells mainly causes the activation of its previously associated target loci and mRNA, i.e., HmgB1 can act as a buffer factor. Approximately ~1/5 of the HmgB1-associated positions are located at the TAD border. Most of these HmgB1-labeled TADs contain SASP-associated genes that are activated when the cell enters senescence. At the same time, a low content of HmgB1 in the nucleus is necessary for the full development of SASP [181]. This is due to the fact that there is a need to mitigate the regulatory effects that HmgB1 has on active promoters, on mRNA, and also on TAD boundaries.

This is a rare example of a regulatory chain where programmed deregulation in the nucleus is directly and quantitatively cross-acting with signaling in the extracellular space to initiate paracrine activation. Thus, the senescent regulatory program is highly dependent on nuclear events prior to the onset of the SASP phenotype.

## 7. Conclusions

Non-histone protein HmgB1 plays important roles in functioning of eukaryotic cell. The functional variety of HmgB1 is associated with the redox state of the protein and combination of various PTMs. On the other hand, HmgB1 is a regulatory protein of chromatin, which takes part in various cellular processes, such as DNA repair, transcription, and recombination. Nuclear HmgB1 protein is an important architectural factor that acts as chaperone of DNA. However, this protein can also move from the nucleus to the cytoplasm, followed by secretion into the extracellular space. Loss of nuclear localization of HmgB1 leads to genome instability with telomere shortening [145], which is the main reason of oncogenesis. Deficiency in HmgB1 in the cell nucleus leads to dysfunction of autophagy and can induce inflammation, which also contributes to tumorigenesis. At the same time, the protein itself, as soon as it goes outside the nucleus, acquires completely different functions, and is characterized by a different redox state and post-translational modifications. 

Moreover, the action of the extra-nuclear protein HmgB1 is also highly diverse (Table 1). On the one hand, it promotes wound healing, tissue regeneration, and on the other hand, it takes part in the initiation of the immune response. It has been shown that HmgB1 is involved in many inflammatory processes, where it acts according to more or less the same principle: the protein enters to the extracellular space, activating inflammatory cascades through its receptors (questions about the effect of the HmgB1 protein on inflammatory processes, oncological, cardiovascular, and neurodegenerative diseases and other pathologies are discussed in more detail in reviews [52,152,153]). In animal experiments, it was demonstrated that inhibition of extracellular HmgB1 attenuates inflammation and increase the organism defenses against several diseases (including sepsis [210], diabetes [211], ischemia, and damage to the heart and liver [212,213]). Approaches to preventing, relieving, or suppressing inflammation, involving the use of antibodies to HmgB1 protein, appear promising [214,215,216]. The indisputable advantage of this approach is that it can be used to treat of patients or alleviate of their condition for which immunosuppressive therapy is contraindicated (for example, due to an additional viral infection). At the same time, since inflammation accompanies other diseases, suppression of inflammation by HmgB1 might allow the organism to concentrate on the underlying disease itself and successfully cope with it. This probably allows the organism to concentrate on the underlying disease and cope with it. In most cases of carcinomas, significantly increased levels of HMGB-domain proteins are observed directly within the cancer tissue, as well as a significant increase in proliferation and, accordingly, tumor growth are observed, revealing anti-apoptotic pro-oncogenic function of HmgB1. On the other hand, proteins of this family, in the absence of inflammation in the organism, can serve as markers of some cancers. However, there is currently no information about HMGB’s role in such carcinomas as lung cancer, blood cancer, and some others. Analysis of these carcinomas will provide a more complete picture of the possible role of HMGB-domain proteins in cancer.

Thus, the functional role, expression level, modification status, redox status, and localization of HmgB1 are all subject to further studies, which, from our prospective, are of great importance with the regard to treatment of multiple diseases [52,151,153,217], including COVID-19 [218]. It was demonstrated very recently that HmgB1, acting inside cells as chromatin regulator, rather than outside cells as an alarmin or chemokine, regulates SARS-CoV-2 infection. It was shown that HmgB1 regulates the receptor ACE2 expression, which is one of the SARS-CoV-2 host factors, and is critical for entry of SARS-CoV-2, SARS-CoV-1, and NL63 viruses [218]. However, detailed mechanism of HmgB1 role in viral infection remains to be determined. One can consider HmgB1 protein as a factor that regulates susceptibility to such coronaviruses such as SARS-CoV-2, which is becoming today a priority topic of molecular biology and medicine research.

## Figures and Tables

**Table 1:** Interactions of HmgB1 with other proteins.

Localization of Protein	Partner	Functions
Nuclear HmgB1	Linker histone H1	C-terminal sequence of HmgB1 binds N-terminal region of H1, disrupting its interactions with DNA and leading to the displacement of H1 [29,30] to facilitate the sliding of nucleosome along the DNA, which is essential for the binding of transcription factors [12,72,83].
	Transcription factors	The interaction of transcription factors with DNA might occur via the formation of an intermediate complex TF1–DNA–HmgB1, whose formation induces attachment of other regulatory chromatin proteins [11].
	Hsp70, MSH2, MLH1	HmgB1 works during the initial stages of recognition/damage of the DNA and interacts with MMR proteins, [113,114,115].
	Enzymes which take part in BER and NER	Base excision repair (BER) and nucleotide excision repair (NER) are also associated with the interaction between HmgB1 and various enzymes [67,68,69,70,116,117,118].
	DNA-dependent protein kinase RAG1 and RAG2 proteins T4 DNA ligase	Repair of double-strand breaks (DSBR) involves DNA-dependent protein kinase (DNA-PKcs) [119]: in vitro HmgB1 protein stimulates activity of DNA-Pkcs [119], RAG1, and RAG2 proteins [120] and increases the activity of T4 DNA ligase [121]. The complex between RAG1, RAG2, and HmgB1 proteins induces the formation of hairpin on DNA [122].
	Tumor suppressor p53 protein	Interaction of p53 with the A-domain of HmgB1 is regulated by C-terminal tail of HmgB1 [131]. HmgB1/p53 complex regulates apoptosis and autophagy [135,136].
	Nuclear factor (NF)-κB Steroid hormone receptors Glucocorticoid receptors	HmgB1 regulates the transcriptional activity of these proteins [133,160].
	Nuclear export protein CRM1	N-glycosylation of HmgB1 is important for binding with CRM1 [85].
	LPS (lipopolysaccharide)	N-glycosylation of HmgB1 is mediated by phorbol 12-myristate 13-acetate (PMA), trichostatin A (TSA) and lipopolysaccharide (LPS) and can lead to the secretion of the protein into the extracellular space as a result of decreasing HmgB1-DNA binding affinity and increasing association with nuclear export protein CRM1 [85].
	cPKC (calcium/phospholipid-dependent protein kinase C)	In vertebrates, phosphorylation of HmgB1 involves calcium/phospholipid-dependent protein kinase C (cPKC) by the PI3K-PKC signaling pathway [77].
	Phosphatase inhibitors (TNF-α or okadaic acid)	HmgB1 can be phosphorylated in mouse macrophage cells RAW264.7 and human monocytes after their treatment with these phosphatase inhibitors, leading to HmgB1 translocation to cytoplasm with possible subsequent secretion into the extracellular space [75].
	PARP1 (poly-ADP-ribose polymerase 1)	PARP1 promotes repair of damaged bases and single-stranded DNA breaks by modulating the structure of chromatin and binding DNA repair factors [89]. Thus, there is a cross-link between ADP-ribosylation of HmgB1 and PARP1 in regulating cell death.
Extranuclear HmgB1	RAGE (receptor for advanced glycation end products)	ADP-ribosylation affects the binding of HmgB1 to RAGE [88]. fr-HmgB1 can bind to RAGE and promote autophagy by inhibiting mTOR kinase and promoting Beclin1–Ptdlns3KC3 complex formation [171]. RAGE binding to fr-HmgB1 stimulates production of CXCL12. ds-HmgB1–RAGE interaction leads to activation of neutrophils and the formation of their extracellular traps [164,165].
	Toll-like receptor TLR9	HmgB1 interacts with TLR9, increasing cytokine production [168].
	Toll-like receptors TLR2 and TLR4	ds-HmgB1/TLR4-MD2 complex stimulates release of inflammatory and angiogenic factors by activation of transcription nuclear factor NF-κB. The interaction of HmgB1 with TLR2 or TLR4 regulates inflammation process when lungs or liver are damaged due to epilepsy, heart disease, or cancer [52].
	Toll-like receptor TLR5	The HmgB1–TLR5 complex activates the NF-κB signaling pathway through the adapter protein MyD88, involved in signal transmission from toll-like receptors, which leads to increased synthesis of pro-inflammatory cytokines [152].
	TIM-3 (T cell immunoglobulin mucin-3)	The interaction of HmgB1 with TIM-3 inhibits the activity of dendritic cells [152].
	Rap1 (Ras-associated protein-1)	HmgB1 activates signal pathway of Rap1 [185].
	TLR9	HmgB1 interacts with TLR9 of endoplasmic reticulum and Golgi complex [152].
	Illexin IL-6	The oxidized form of the HMGB1 protein stimulates the secretion of pro-inflammatory cytokines, including IL-6 by activation of the TLR-4 receptor [152].
